# Supplementary material for: Inactivation of LATS1/2 drives luminal-basal plasticity to initiate basal-like mammary carcinomas
Source: Nat Commun. 2022 Nov 28;13:7198. doi: 10.1038/s41467-022-34864-8 (PMC9705439; doi:10.1038/s41467-022-34864-8)
Supplement: Supplementary file 1 — Supplementary Information [file 41467_2022_34864_MOESM1_ESM.pdf]

Supplementary Information for

Inactivation of LATS1/2 drives luminal-basal plasticity to initiate basal-like mammary carcinomas

Joseph G Kern, Andrew M Tilston-Lunel, Anthony Federico, Boting Ning, Amy Mueller, Grace B Peppler, Eleni Stampouloglou, Nan Cheng, Randy L Johnson, Marc E Lenburg, Jennifer E Beane, Stefano Monti, Xaralabos Varelas

Corresponding author: Xaralabos Varelas
Email: xvarelas@bu.edu

This PDF file includes:

Supplementary Fig. 1
Legend for Supplementary Fig. 1

Supplementary Fig. 2
Legend for Supplementary Fig. 2

Supplementary Fig. 3
Legend for Supplementary Fig. 3

Supplementary Fig. 4
Legend for Supplementary Fig. 4

Supplementary Table 1
Supplementary Table 2
Supplementary Table 3

Other supplementary materials for this manuscript include the following:

Supplementary Data 1
Supplementary Data 2
Supplementary Data 3
Supplementary Data 4

Sup Fig. 1

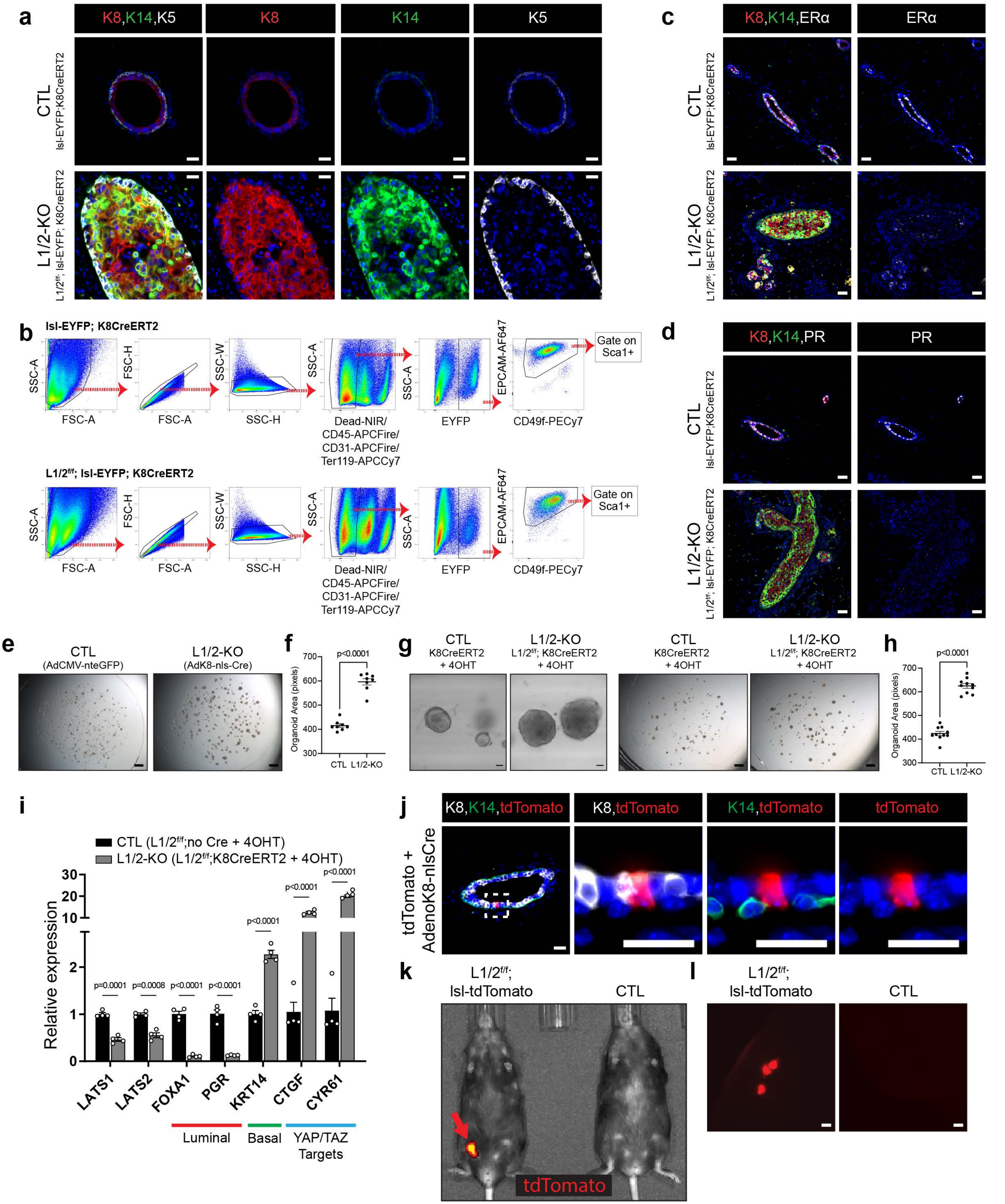
a
K8,K14,K5
K8
K14
K5
CTL
lsl-EYFP;K8CreERT2
L1/2-KO
L1/2f/f; lsl-EYFP; K8CreERT2
b
lsl-EYFP; K8CreERT2
L1/2f/f; lsl-EYFP; K8CreERT2
SSC-A
FSC-A
FSC-H
SSC-W
SSC-H
Dead-NIR/
CD45-APCFire/
CD31-APCFire/
Ter119-APCCy7
EYFP
EPCAM-AF647
CD49f-PECy7
Gate on Sca1+
c
K8,K14,ERα
ERα
d
K8,K14,PR
PR
e
CTL
(AdCMV-nteGFP)
L1/2-KO
(AdK8-nls-Cre)
f
Organoid Area (pixels)
p<0.0001
CTL
L1/2-KO
g
CTL
K8CreERT2
+ 4OHT
L1/2-KO
L1/2f/f; K8CreERT2
+ 4OHT
h
i
CTL (L1/2f/f;no Cre + 4OHT)
L1/2-KO (L1/2f/f;K8CreERT2 + 4OHT)
Relative expression
p=0.0001
p=0.0008
p<0.0001
LATS1
LATS2
FOXA1
PGR
KRT14
CTGF
CYR61
Luminal
Basal
YAP/TAZ Targets
j
K8,K14,tdTomato
K8,tdTomato
K14,tdTomato
tdTomato
tdTomato +
AdenoK8-nlsCre
k
L1/2f/f;
lsl-tdTomato
CTL
tdTomato
l
L1/2f/f;
lsl-tdTomato
CTL

Supplementary Fig. 1. **Loss of LATS1/2 in luminal mammary cells induces mammary carcinomas with luminal-basal plasticity.** **(A)** IF of K8, K14, and K5 in control and Lats1/2$^{f/f}$;K8CreERT2 mammary ducts (Scale bar, 20 μM). **(B)** Flow cytometric gating strategy used to examine control and Lats1/2$^{f/f}$;K8CreERT2 mammary epithelial cells. **(C)** IF of K8, K14, and ERα in control and Lats1/2$^{f/f}$;K8CreERT2 mammary ducts (Scale bar, 50 μM). **(D)** IF of K8, K14, and PR in control and Lats1/2$^{f/f}$;K8CreERT2 mammary ducts (Scale bar, 50 μM). **(E)** Morphology of organoids cultured from control and Lats1/2$^{f/f}$ mice infected with AdK8-nls-Cre (Scale bar, 1 mm). **(F)** Quantification of average individual organoid area measured per well in control (AdCMV-nteGFP) (CTL) and Lats1/2$^{f/f}$ (L1/2-KO) organoids infected with AdK8-nls-Cre (n=8 per condition. Unpaired two-tailed t-test. Data are shown with mean ± SEM). **(G)** Morphology of organoids cultured from control and Lats1/2$^{f/f}$;K8CreERT2 mice treated with 4OHT (high magnification on the left with Scale bar, 50 μM, low magnification on the right with Scale bar, 1 mm). **(H)** Quantification of average individual organoid area measured per well in organoids cultured from control (CTL) and Lats1/2$^{f/f}$;K8CreERT2 (L1/2-KO) mice treated with 4OHT (n=10 per condition. Unpaired two-tailed t-test. Data are shown with mean ± SEM). **(I)** Expression of selected luminal and basal/stem-cell markers in control and Lats1/2$^{f/f}$;K8CreERT2 organoids treated with 4OHT for 7 days (n=4. Unpaired two-tailed t-tests. Data are shown as mean ± SEM). **(J)** IF of K8, K14, and tdTomato in the mammary epithelium of a control lsl-tdTomato mouse injected with AdK8-nls-Cre (n=1) (Scale bar, 20 μM). **(K)** IVIS live imaging of a primary tumor formed in the mammary gland of a Lats1/2$^{f/f}$;lsl-tdTomato mouse approximately 13 months after injection with AdK8-nls-Cre. **(L)** Imaging of tdTomato+ cells that have metastasized to the lungs of a Lats1/2$^{f/f}$;lsl-tdTomato mouse approximately 13 months after injection with AdK8-nls-Cre (n=2) (Scale bar, 50 μM). Source data are provided as a source data file.

Sup Fig. 2

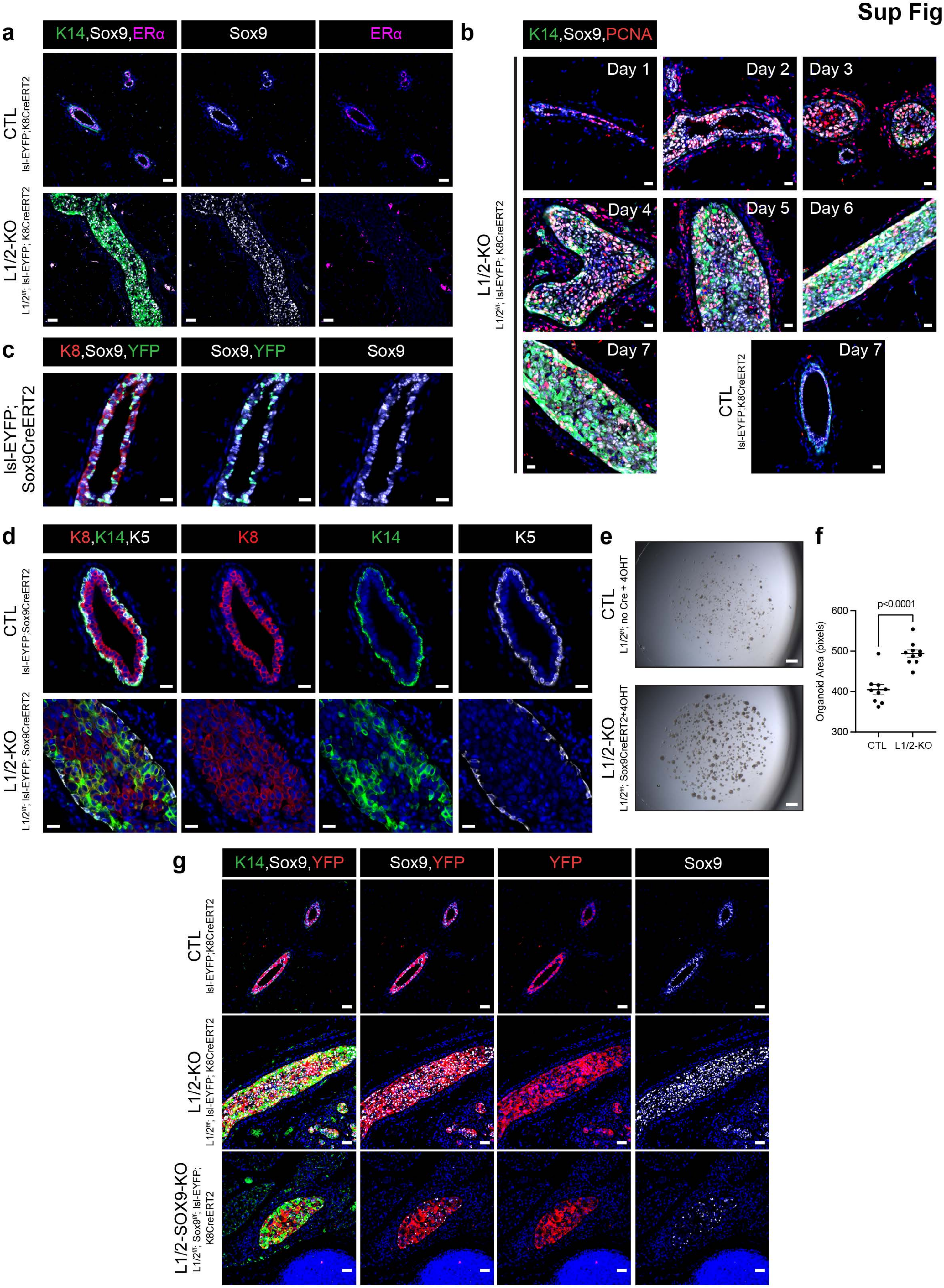
a
K14,Sox9,ERα
Sox9
ERα
CTL
lsl-EYFP;K8CreERT2
L1/2-KO
L1/2f/f; lsl-EYFP; K8CreERT2
b
K14,Sox9,PCNA
Day 1
Day 2
Day 3
Day 4
Day 5
Day 6
Day 7
Day 7
L1/2-KO
L1/2f/f; lsl-EYFP; K8CreERT2
CTL
lsl-EYFP;K8CreERT2
c
K8,Sox9,YFP
Sox9,YFP
Sox9
lsl-EYFP;
Sox9CreERT2
d
K8,K14,K5
K8
K14
K5
CTL
lsl-EYFP;Sox9CreERT2
L1/2-KO
L1/2f/f; lsl-EYFP; Sox9CreERT2
e
CTL
L1/2f/f; no Cre + 4OHT
L1/2-KO
L1/2f/f; Sox9CreERT2+4OHT
f
p<0.0001
Organoid Area (pixels)
600
500
400
300
CTL
L1/2-KO
g
K14,Sox9,YFP
Sox9,YFP
YFP
Sox9
CTL
lsl-EYFP;K8CreERT2
L1/2-KO
L1/2f/f; lsl-EYFP; K8CreERT2
L1/2-SOX9-KO
L1/2f/f; Sox9f/f; lsl-EYFP;
K8CreERT2

Supplementary Fig. 2. Sox9 expressing luminal cells serve as cells of origin for LATS1/2-null carcinomas. (A) IF of K14, Sox9, and ERα in control and Lats1/2$^{f/f}$;K8CreERT2 mammary ducts (Scale bar, 50 μM). **(B)** IF of K14, Sox9, and PCNA in control and Lats1/2$^{f/f}$;K8CreERT2 mammary ducts collected 1-7 days after the last Tamoxifen dose (n=2 each) (Scale bar, 20 μM). **(C)** IF of K8, Sox9, and YFP in lsl-EYFP;Sox9CreERT2 mammary epithelia (Scale bar, 20 μM) (n=2). **(D)** IF of K8, K14, and K5 in control and Lats1/2$^{f/f}$;lsl-EYFP;Sox9CreERT2 mammary ducts (Scale bar, 20 μM) (n=2 for CTL). **(E)** Morphology of organoids cultured from control and Lats1/2$^{f/f}$; Sox9CreERT2 mice treated with 4OHT (Scale bar, 1 mm). **(F)** Quantification of average individual organoid area measured per well in organoids cultured from control (CTL) and Lats1/2$^{f/f}$; Sox9CreERT2 (L1/2-KO) mice treated with 4OHT (n=9 CTL, n=10 L1/2-KO. Unpaired two-tailed t-test. Data are shown with mean ± SEM) **(G)** IF of K14, YFP, and Sox9 in control, Lats1/2$^{f/f}$;lsl-EYFP;K8CreERT2, and Lats1/2$^{f/f}$;Sox9f$^{f/f}$; lsl-EYFP;K8CreERT2 mammary ducts (Scale bar, 50 μM). Source data are provided as a source data file.

Sup Fig. 3

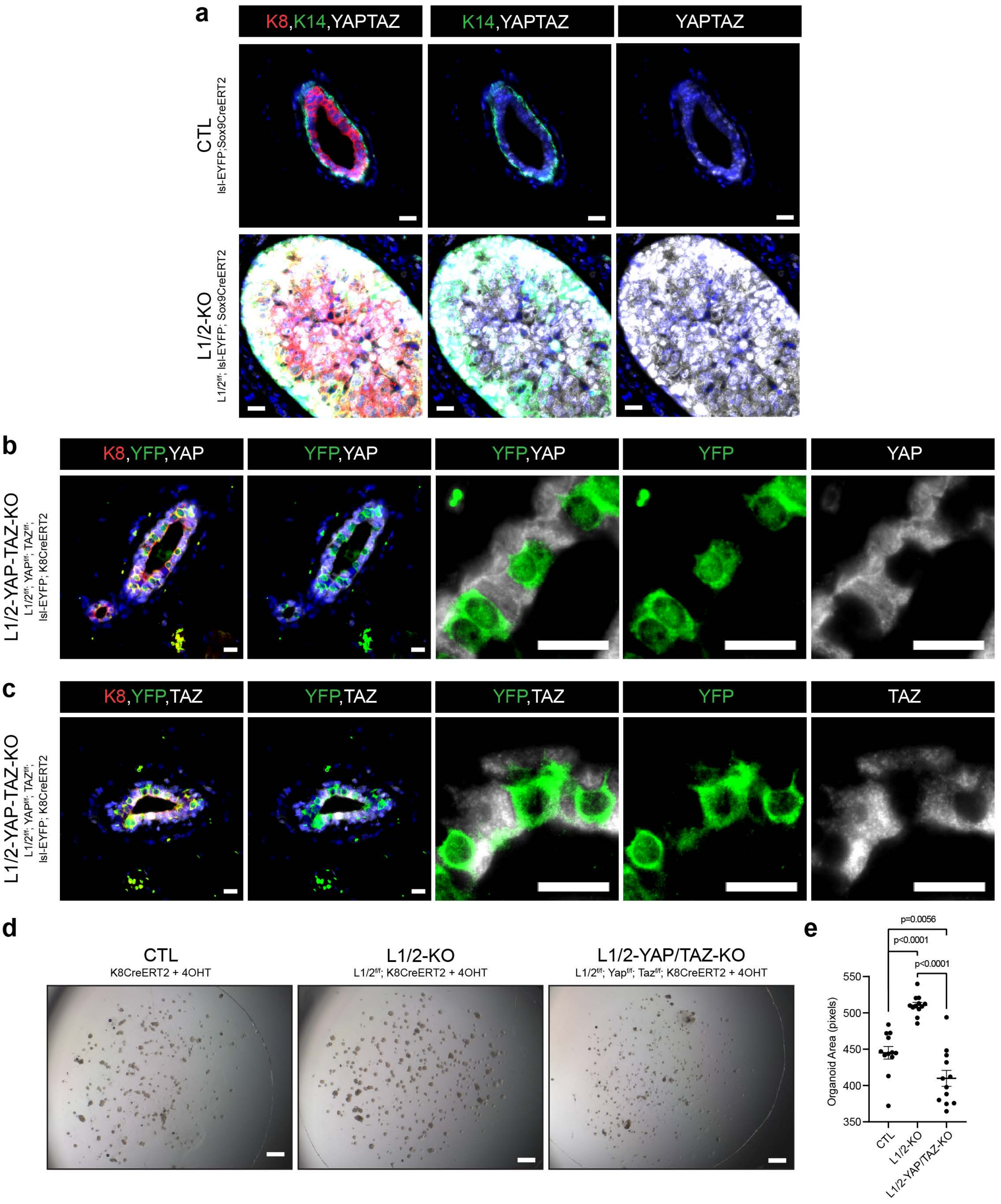
a
K8,K14,YAPTAZ
K14,YAPTAZ
YAPTAZ
CTL
lsl-EYFP;Sox9CreERT2
L1/2-KO
L1/2f/f; lsl-EYFP; Sox9CreERT2
b
K8,YFP,YAP
YFP,YAP
YFP,YAP
YFP
YAP
L1/2-YAP-TAZ-KO
L1/2f/f; YAPf/f; TAZf/f;
lsl-EYFP; K8CreERT2
c
K8,YFP,TAZ
YFP,TAZ
YFP,TAZ
YFP
TAZ
L1/2-YAP-TAZ-KO
L1/2f/f; YAPf/f; TAZf/f;
lsl-EYFP; K8CreERT2
d
CTL
K8CreERT2 + 4OHT
L1/2-KO
L1/2f/f; K8CreERT2 + 4OHT
L1/2-YAP/TAZ-KO
L1/2f/f; Yapf/f; Tazf/f; K8CreERT2 + 4OHT
e
p=0.0056
p<0.0001
p<0.0001
Organoid Area (pixels)
550
500
450
400
350
CTL
L1/2-KO
L1/2-YAP/TAZ-KO

Supplementary Fig. 3. Mammary carcinomas driven by LATS1/2 loss are dependent on YAP and TAZ. (A) IF of K8, K14 and YAP/TAZ in control and Lats1/2$^{f/f}$;lsl-EYFP;Sox9CreERT2 mammary glands (Scale bar, 20 μM) (n=2 for CTL). **(B)** IF of YAP, K8 and YFP in LATS1/2$^{f/f}$;YAP$^{f/f}$;TAZ$^{f/f}$;lsl-EYFP;K8CreERT2 mammary ducts (Scale bar, 20 μM). **(C)** IF of TAZ, K8, and YFP in LATS1/2$^{f/f}$;YAP$^{f/f}$;TAZ$^{f/f}$;lsl-EYFP;K8CreERT2 mammary ducts (Scale bar, 20 μM). **(D)** Morphology of organoids cultured from control, LATS1/2$^{f/f}$;K8CreERT2, and LATS1/2$^{f/f}$;YAP$^{f/f}$;TAZ$^{f/f}$;K8CreERT2 mice treated with 4OHT (Scale bar, 1 mm). **(E)** Quantification of average individual organoid area measured per well of organoids cultured from control (CTL), LATS1/2$^{f/f}$;K8CreERT2 (L1/2-KO), and LATS1/2$^{f/f}$;YAP$^{f/f}$;TAZ$^{f/f}$;K8CreERT2 (L1/2-YAP/TAZ-KO) mice (n=12 per condition. One-way ANOVA with Fisher's least significant difference multiple comparisons test. Data are shown with mean ± SEM). Source data are provided as a source data file.

Sup Fig. 4

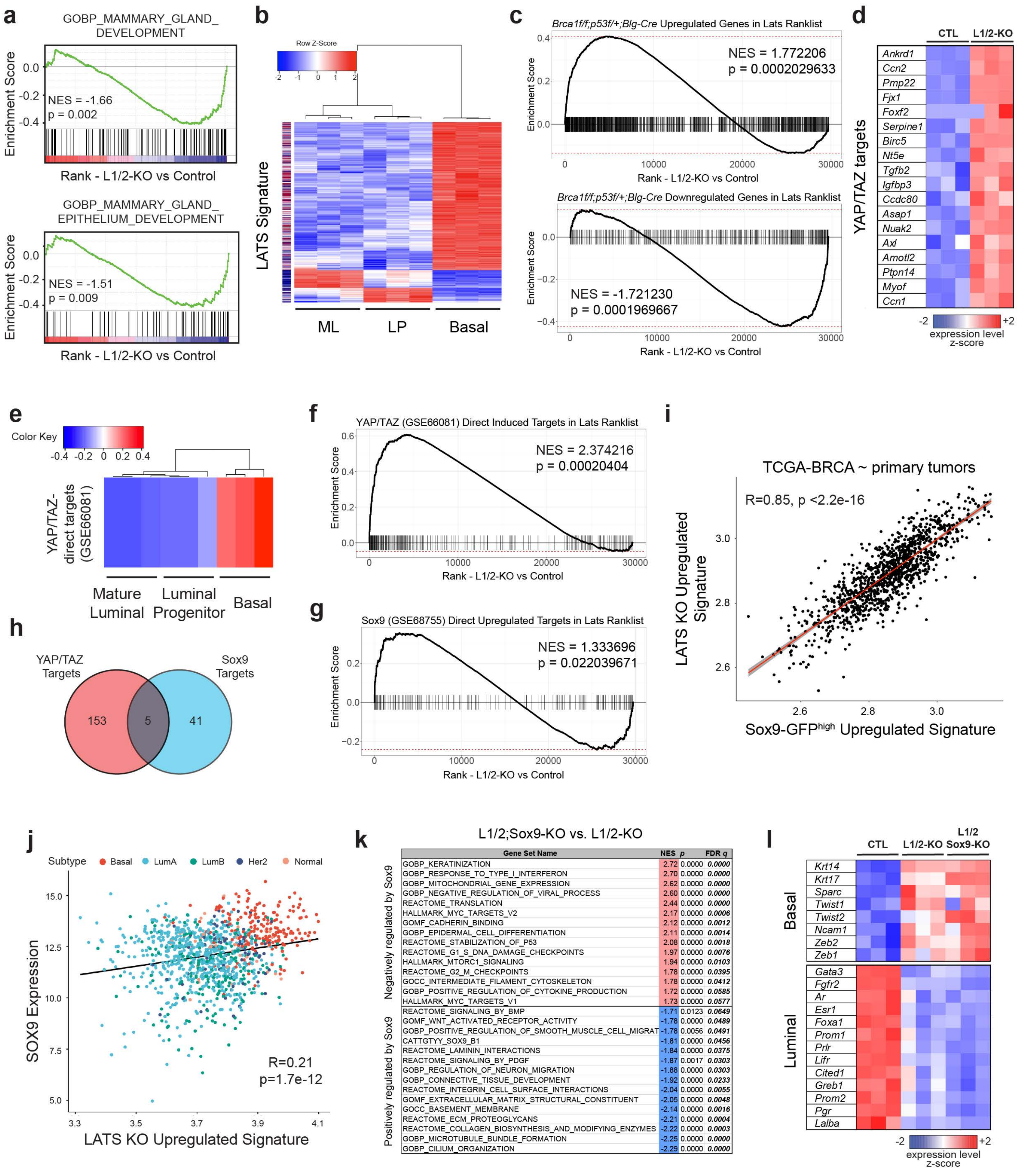

Gene Set Name	NES	*p*	FDR *q*
GOBP_KERATINIZATION	2.72	0.0000	***0.0000***
GOBP_RESPONSE_TO_TYPE_I_INTERFERON	2.70	0.0000	***0.0000***
GOBP_MITOCHONDRIAL_GENE_EXPRESSION	2.62	0.0000	***0.0000***
GOBP_NEGATIVE_REGULATION_OF_VIRAL_PROCESS	2.60	0.0000	***0.0000***
REACTOME_TRANSLATION	2.44	0.0000	***0.0000***
HALLMARK_MYC_TARGETS_V2	2.17	0.0000	***0.0006***
GOMF_CADHERIN_BINDING	2.12	0.0000	***0.0012***
GOBP_EPIDERMAL_CELL_DIFFERENTIATION	2.11	0.0000	***0.0014***
REACTOME_STABILIZATION_OF_P53	2.08	0.0000	***0.0018***
REACTOME_G1_S_DNA_DAMAGE_CHECKPOINTS	1.97	0.0000	***0.0076***
HALLMARK_MTORC1_SIGNALING	1.94	0.0000	***0.0103***
REACTOME_G2_M_CHECKPOINTS	1.78	0.0000	***0.0395***
GOCC_INTERMEDIATE_FILAMENT_CYTOSKELETON	1.78	0.0000	***0.0412***
GOBP_POSITIVE_REGULATION_OF_CYTOKINE_PRODUCTION	1.72	0.0000	***0.0585***
HALLMARK_MYC_TARGETS_V1	1.73	0.0000	***0.0577***
REACTOME_SIGNALING_BY_BMP	-1.71	0.0123	***0.0649***
GOMF_WNT_ACTIVATED_RECEPTOR_ACTIVITY	-1.78	0.0000	***0.0489***
GOBP_POSITIVE_REGULATION_OF_SMOOTH_MUSCLE_CELL_MIGRAT	-1.78	0.0056	***0.0491***
CATTGTYY_SOX9_B1	-1.81	0.0000	***0.0456***
REACTOME_LAMININ_INTERACTIONS	-1.84	0.0000	***0.0375***
REACTOME_SIGNALING_BY_PDGF	-1.87	0.0017	***0.0303***
GOBP_REGULATION_OF_NEURON_MIGRATION	-1.88	0.0000	***0.0303***
GOBP_CONNECTIVE_TISSUE_DEVELOPMENT	-1.92	0.0000	***0.0233***
REACTOME_INTEGRIN_CELL_SURFACE_INTERACTIONS	-2.04	0.0000	***0.0055***
GOMF_EXTRACELLULAR_MATRIX_STRUCTURAL_CONSTITUENT	-2.05	0.0000	***0.0048***
GOCC_BASEMENT_MEMBRANE	-2.14	0.0000	***0.0016***
REACTOME_ECM_PROTEOGLYCANS	-2.21	0.0000	***0.0004***
REACTOME_COLLAGEN_BIOSYNTHESIS_AND_MODIFYING_ENZYMES	-2.22	0.0000	***0.0003***
GOBP_MICROTUBULE_BUNDLE_FORMATION	-2.25	0.0000	***0.0000***
GOBP_CILIUM_ORGANIZATION	-2.29	0.0000	***0.0000***

<u>Supplementary Fig. 4</u>. Luminal LATS1/2 loss promotes basal-like and YAP/TAZ-enriched transcriptional programs, and comparisons between LATS1/2-null and Sox9-associated signatures. (A) Selected GSEA-generated gene sets enriched in the downregulated signatures of LATS1/2- null cells compared to control. **(B)** Heatmap of LATS-upregulated and LATS-downregulated genes against previously identified signatures of normal mammary luminal mature, luminal progenitor, and basal cells (GSE63310). For the left side column, dark red indicates a gene upregulated with LATS deletion, dark blue indicates a gene downregulated with LATS deletion, and white indicates a gene not differentially expressed with LATS deletion. **(C)** GSEA analysis comparing signatures of LATS1/2-null cells to upregulated and downregulated signatures derived from the *Brca1f/f; p53f/+; Blg-Cre* mouse model of basal-like breast cancer. **(D)** Expression of selected YAP/TAZ-target genes in LATS1/2-null cells relative to control as colored by variance-stabilizing transformation (VST). **(E)** GSVA analysis comparing YAP/TAZ direct induced target genes (GSE66081) to signatures of normal mammary luminal mature, luminal progenitor, and basal cells (GSE63310). **(F)** GSEA comparing the signature of LATS1/2-null cells to YAP/TAZ direct induced target genes (GSE66081). **(G)** GSEA comparing the signature of LATS1/2-null cells to Sox9 direct induced target genes (GSE68755). **(H)** Comparison of leading-edge genes identified in **(F)** and **(G)**. **(I)** ssGSEA analysis of the LATS-upregulated signature against the Sox9-GFP^{high}-upregulated signature in human breast cancers (n=1102. Two-sided Pearson correlation. No adjustment for multiple comparisons). **(J)** ssGSEA analysis of the LATS-upregulated signature against Sox9 expression levels in human breast cancers colored by subtype (Two-sided Pearson correlation. No adjustment for multiple comparisons). **(K)** Selected GSEA-generated genesets enriched in the upregulated (red) and downregulated (blue) signatures of LATS1/2-Sox9-null cells compared to LATS1/2-null cells. **(L)** Relative expression of selected luminal and basal-associated genes identified in EYFP+ cells isolated from lsl-EYFP;K8CreERT2, LATS1/2$^{f/f}$;lsl-EYFP;K8CreERT2mice, and LATS1/2$^{f/f}$; Sox9$^{f/f}$;lsl-EYFP;K8CreERT2 mice as analyzed via RNA-sequencing and colored by VST (p>0.05 for rescue in L1/2-KO versus L1/2;Sox9-null). For all GSEA rank-list plots, nominal uncorrected p-values are shown.

Supplementary Table 1. Mice

Strain	Alias	Source	Stock
Lats1tm1.1Jfm/RjoJ	LATS1$^{f/f}$	The Jackson Laboratory	#024941
Lats2tm1.1Jfm/RjoJ	LATS2$^{f/f}$	The Jackson Laboratory	#025428
STOCK Tg(Krt8-cre/ERT2)17Blpn/J	Krt8-CreERT2	The Jackson Laboratory	#017947
B6.129X1-Gt(ROSA)26Sortm1(EYFP)Cos/J	R26-LSL-EYFP	The Jackson Laboratory	#006148
Wwtr1^{tm1Hmc} Yap1^{tm1Hmc}/WranJ	YAP1$^{f/f}$; WWTR1$^{f/f}$	The Jackson Laboratory	# 030532
Sox9-Cre (B6.129S7-Sox9<tm1(cre/ERT2)Haak>)	Sox9-CreERT2	RIKEN	RBRC05522
B6.129S7-Sox9tm2Crm/J	Sox9$^{f/f}$	The Jackson Laboratory	#013016
B6.Cg-Gt(ROSA)26Sortm14(CAG-tdTomato)Hze/J	LSL-tdTomato	The Jackson Laboratory	#007914

Supplementary Table 2. Primary and Secondary Antibodies

Target	Species	Company	Catalogue #	Application	Dilution
Keratin 8	Rat	DHSB	TROMA-1c	IF	1:200
Keratin 14 – FITC	Mouse	Millipore Sigma	CBL197F	IF	1:20
Keratin 14	Rabbit	Biolegend	905301	IF	1:400
Keratin 5	Rabbit	Biolegend	905501	IF	1:400
Phospho-LATS1/2 (Thr1079/1041)	Rabbit	Assay BioTech	A8125	IF	1:100
YAP/TAZ	Rabbit	CST	8418	IF	1:100
GFP/YFP	Chicken	Aves Labs, Inc.	GFP-1020	IF	1:400
GFP/YFP	Rabbit	Takara Bio Clontech	632375	IF	1:100
ERα	Rabbit	Abcam	Ab32063	IF	1:100
PR	Rabbit	Santa Cruz	SC-538	IF	1:100
Sox9	Goat	R&D Systems	AF3075	IF	1:100
Sox9	Rabbit	Millipore-Sigma	AB5535	IF	1:100
YAP	Rabbit	CST	D8H1X	IF	1:100
TAZ	Rabbit	CST	E8E9G	IF	1:100
tdTomato	Goat	Sicgen	AB8181-200	IF	1:100
PCNA	Mouse	CST	PC10	IF	1:200
CD45 – APC/Fire	Rat	Biolegend	103153	FC	1:400
CD31 – APC/Fire	Rat	Biolegend	102433	FC	1:50
Ter119 – APC/Cy7	Rat	Biolegend	116223	FC	1:100
EpCAM – Alexa 647	Rat	Biolegend	118211	FC	1:800
CD49f – PE/Cy7	Rat	Biolegend	313621	FC	1:800
CD49b – PE	Hamster	Biolegend	103506	FC	1:800
Sca-1 – BV421	Rat	Biolegend	108127	FC	1:800
Donkey α-Rabbit Cy3		Jackson ImmunoResearch	711-166-152	IF	1:400
Donkey α-Rabbit 647		Jackson ImmunoResearch	711-606-152	IF	1:400
Donkey α-Goat 647		Jackson ImmunoResearch	705-605-147	IF	1:400
Donkey α-Rat 488		Jackson ImmunoResearch	712-546-153	IF	1:500
Donkey α-Rat 594		Jackson ImmunoResearch	712-586-153	IF	1:400
Donkey α-Rat Cy3		Jackson ImmunoResearch	712-165-153	IF	1:400
Donkey α-Chicken 594		Jackson ImmunoResearch	703-585-155	IF	1:400
Donkey α-Chicken 647		Jackson ImmunoResearch	703-605-155	IF	1:400
Donkey α-Mouse 647		Jackson ImmunoResearch	715-606-150	IF	1:400

Supplementary Table 3. qRT-PCR Primers Used

Target	Species	Forward Primer	Reverse Primer
PPIA	Mouse	GAGCTGTTTGCAGACAAAGTTC	CCCTGGCACATGAATCCTGG
LATS1	Mouse	CCATGAAACCAGGAAATGTGCA	CTAGAGATGGGCCGTGTCTC
LATS2	Mouse	GCTACAGCTGGAGCAGGAAA	CCCTCTTCAGCCGGTTGTAG
YAP	Mouse	AGCTGCCCGACTCCTTCT	CGAACATGCTGTGGAGTCAG
WWTR1	Mouse	CAGCCTCTGAATCATGTGAACCTC	GGCTAGTGGCCACGACTTGCTGGT
FOXA1	Mouse	ATGAGAGCAACGACTGGAACA	TCATGGAGTTCATAGAGCCCA
PGR	Mouse	GCCTGACACTTCCAGCTCTT	CCGGAAACCTGGCAGAGATT
KRT14	Mouse	AGCGGCAAGAGTGAGATTTCT	CCTCCAGGTTATTCTCCAGGG
SOX9	Mouse	AGTACCCGCATCTGCACAAC	ACGAAGGGTCTCTTCTCGCT
CD44	Mouse	GGCTCTGATTCTTGCCGTCT	TGTCTTCCACCGTCCCATTG
CTGF	Mouse	AGACCTGTGGGATGGGCAT	GCTTGGCGATTTTAGGTGTCC
CYR61	Mouse	TAAGGTCTGCGCTAAACAACTC	CAGATCCCTTTCAGAGCGGT
