# Supplementary material for: Inactivation of LATS1/2 drives luminal-basal plasticity to initiate basal-like mammary carcinomas
Source: Nat Commun. 2022 Nov 28;13:7198. doi: 10.1038/s41467-022-34864-8 (PMC9705439; doi:10.1038/s41467-022-34864-8)
Supplement: Supplementary file 3 — Description of Additional Supplementary Files [file 41467_2022_34864_MOESM3_ESM.pdf]

Description of Additional Supplementary Files

File Name: Supplementary Data 1
Description: Differential gene expression data

File Name: Supplementary Data 2
Description: GSEA pathway enrichment analysis of L1/2-KO gene signature

File Name: Supplementary Data 3
Description: YAP/TAZ Leading Edge Targets in LATS Ranklist

File Name: Supplementary Data 4
Description: Binding Analysis for Regulation of Transcription (BART) of the top 500 genes upregulated in LATS1/2-K8 deleted cells
