# Supplementary material for: Inactivation of LATS1/2 drives luminal-basal plasticity to initiate basal-like mammary carcinomas
Source: Nat Commun. 2022 Nov 28;13:7198. doi: 10.1038/s41467-022-34864-8 (PMC9705439; doi:10.1038/s41467-022-34864-8)
Supplement: Supplementary file 8 — Reporting Summary [file 41467_2022_34864_MOESM8_ESM.pdf]

nature portfolio

Corresponding author(s): Xaralabos Varelas
Last updated by author(s): Feb 17, 2022

Reporting Summary

Nature Portfolio wishes to improve the reproducibility of the work that we publish. This form provides structure for consistency and transparency in reporting. For further information on Nature Portfolio policies, see our Editorial Policies and the Editorial Policy Checklist.

Statistics

For all statistical analyses, confirm that the following items are present in the figure legend, table legend, main text, or Methods section.

n/a	Confirmed	
☐	☒	The exact sample size (*n*) for each experimental group/condition, given as a discrete number and unit of measurement
☐	☒	A statement on whether measurements were taken from distinct samples or whether the same sample was measured repeatedly
☐	☒	The statistical test(s) used AND whether they are one- or two-sided<br>*Only common tests should be described solely by name; describe more complex techniques in the Methods section.*
☒	☐	A description of all covariates tested
☐	☒	A description of any assumptions or corrections, such as tests of normality and adjustment for multiple comparisons
☐	☒	A full description of the statistical parameters including central tendency (e.g. means) or other basic estimates (e.g. regression coefficient) AND variation (e.g. standard deviation) or associated estimates of uncertainty (e.g. confidence intervals)
☐	☒	For null hypothesis testing, the test statistic (e.g. *F*, *t*, *r*) with confidence intervals, effect sizes, degrees of freedom and *P* value noted<br>*Give P values as exact values whenever suitable.*
☒	☐	For Bayesian analysis, information on the choice of priors and Markov chain Monte Carlo settings
☒	☐	For hierarchical and complex designs, identification of the appropriate level for tests and full reporting of outcomes
☐	☒	Estimates of effect sizes (e.g. Cohen's *d*, Pearson's *r*), indicating how they were calculated

Our web collection on statistics for biologists contains articles on many of the points above.

Software and code

Policy information about availability of computer code

Data collection	Data was collected on the following instruments: Zeiss LSM 700, Zeiss Axio Observer.Z1, Zeiss Axio Scan.Z1, Zeiss SteREO Discovery.V12, ViiA7 Real-Time PCR System (Applied Biosystems), IVIS Spectrum imaging system (Perkin Elmer), Illumina NextSeq 2000.
Data analysis	The following software and tools were used for data analysis, as described in the methods: GraphPad Prism 9, Zeiss ZEN 3.3 Blue, Zeiss ZEN 3.1 Blue, Zeiss ZEN 2 Blue, Zeiss ZEN 2.3 SP1 FP3 Black, QuantStudio Real-Time PCR Software v1.6.1, FlowJo v10.7.1, FACS Diva 6.2.1, STAR (version 2.6.0c), featureCounts (Subread package, version 1.6.2), SAMtools (version 1.9), FastQC (version 0.11.7), RSeQC (version 3.0.0), DESeq2 R package (version 1.23.10), HomoloGene (version 68), R package ComplexHeatmap (v2.6.2), Gene Set Enrichment Analysis (GSEA) (version 2.2.1), R package GSVA (v1.34.0), Limma-Voom (v3.46), GISTIC 2.0.22 (Firehose task version:140).

For manuscripts utilizing custom algorithms or software that are central to the research but not yet described in published literature, software must be made available to editors and reviewers. We strongly encourage code deposition in a community repository (e.g. GitHub). See the Nature Portfolio guidelines for submitting code & software for further information.

Data

Policy information about availability of data

All manuscripts must include a data availability statement. This statement should provide the following information, where applicable:

- Accession codes, unique identifiers, or web links for publicly available datasets
- A description of any restrictions on data availability
- For clinical datasets or third party data, please ensure that the statement adheres to our policy

TCGA datasets used in the study are publicly available and can be obtained from https://portal.gdc.cancer.gov. Gene expression data used in the study, including new data that was generated, are publicly available on the GEO repository: GSE196555 [https://www.ncbi.nlm.nih.gov/geo/query/acc.cgi?acc=GSE196555],

GSE135885 [https://www.ncbi.nlm.nih.gov/geo/query/acc.cgi?acc=GSE135885], GSE135891 [https://www.ncbi.nlm.nih.gov/geo/query/acc.cgi?acc=GSE135891], GSE63310 [https://www.ncbi.nlm.nih.gov/geo/query/acc.cgi?acc= GSE63310]. The authors declare that all other data supporting this study are either available within the article, supplementary figures, or available from the authors upon request. Source data are provided with this paper.

Field-specific reporting

Please select the one below that is the best fit for your research. If you are not sure, read the appropriate sections before making your selection.

☒ Life sciences ☐ Behavioural & social sciences ☐ Ecological, evolutionary & environmental sciences

For a reference copy of the document with all sections, see nature.com/documents/nr-reporting-summary-flat.pdf

Life sciences study design

All studies must disclose on these points even when the disclosure is negative.

Sample size	Sample sizes were determined based on prior studies performed in the field and the number of replicates for statistical analyses are specified in the figure legends.
Data exclusions	No data were excluded from the statistical analyses. Mice injected with AdK8-nls-Cre were excluded from experimental counts if they died without a confirmation of whether a tumor developed or not.
Replication	The conclusions obtained in this study were made utilizing several transgenic mouse models. The experimental phenotypes observed in LATS1/2f/f;YFP;K8CreERT2, LATS1/2f/f;YFP;Sox9CreERT2, LATS1/2f/f;Sox9f/f;YFP;K8CreERT2, and LATS1/2f/f;YAP f/f;TAZ f/f;YFP;K8CreERT2 mice were all confirmed with successful replication. Lineage traced tumors and metastases that developed in LATS1/2f/f mice injected with AdK8-nls-Cre were successfully replicated with tumors formed at frequencies reported in the text. Validation of AdenoK8-nls-Cre targeting of K8+ cells has been demonstrated in a prior manuscript (PMID: 249036465), which we replicated with a successful injection of AdK8-nls-Cre in a lsl-tdTomato mouse. All qPCR data shown is data collected from an experiment of least three independent wells per condition and is representative of replicate experiments.
Randomization	Randomization was not relevant for this study. Animals were assigned into experimental groups based on their desired genotypes for comparison. Efforts were made to confirm the phenotypes across mice from multiple different litters and cages.
Blinding	Blinding was not conducted as the genotypes of the animals being analyzed in this study were known to the investigators and all statistical analyses were performed using software as described in the methods.

Reporting for specific materials, systems and methods

We require information from authors about some types of materials, experimental systems and methods used in many studies. Here, indicate whether each material, system or method listed is relevant to your study. If you are not sure if a list item applies to your research, read the appropriate section before selecting a response.

Materials & experimental systems

n/a	Involved in the study	
☐	☒	Antibodies
☒	☐	Eukaryotic cell lines
☒	☐	Palaeontology and archaeology
☐	☒	Animals and other organisms
☒	☐	Human research participants
☒	☐	Clinical data
☒	☐	Dual use research of concern

Methods

n/a	Involved in the study	
☒	☐	ChIP-seq
☐	☒	Flow cytometry
☒	☐	MRI-based neuroimaging

Antibodies

Antibodies used

Antibodies used for IHC:
Keratin 8 Rat DHSB TROMA-1c
Keratin 14 – FITC Mouse Millipore Sigma CBL197F Clone LL002
Keratin 14 Rabbit Biolegend 905301 Clone Poly19053
Keratin 5 Rabbit Biolegend 905501 Clone Poly19055
Phospho-LATS1/2 (Thr1079/1041) Rabbit Assay BioTech A8125
YAP/TAZ Rabbit CST 8418
GFP/YFP Chicken Aves Labs, Inc. GFP-1020
GFP/YFP Rabbit Takara Bio Clontech 632592
ERα Rabbit Abcam Ab32063 Clone E115
PR Rabbit Santa Cruz SC-538
Sox9 Goat R&D Systems AF3075

Sox9 Rabbit Millipore-Sigma AB5535
YAP Rabbit CST D8H1X
TAZ Rabbit CST E8E9G
tdTomato Goat Sicgen AB8181-200
PCNA Mouse CST PC10

Antibodies used for flow cytometry:
CD45 – APC/Fire Rat Biolegend 103153 Clone 30-F11
CD31 – APC/Fire Rat Biolegend 102433 Clone 390
Ter119 – APC/Cy7 Rat Biolegend 116223 Clone TER-119
EpCAM – Alexa 647 Rat Biolegend 118211 Clone G8.8
CD49f – PE/Cy7 Rat Biolegend 313621 Clone GoH3
CD49b – PE Hamster Biolegend 103506 Clone Hma2
Sca-1 – BV421 Rat Biolegend 108127 Clone D7

Secondary antibodies:
Donkey α-Rabbit Cy3 Jackson ImmunoResearch 711-166-152
Donkey α-Rabbit 647 Jackson ImmunoResearch 711-606-152
Donkey α-Goat 647 Jackson ImmunoResearch 705-605-147
Donkey α-Rat 488 Jackson ImmunoResearch 712-546-153
Donkey α-Rat 594 Jackson ImmunoResearch 712-586-153
Donkey α-Rat Cy3 Jackson ImmunoResearch 712-165-153
Donkey α-Chicken 594 Jackson ImmunoResearch 703-585-155
Donkey α-Chicken 647 Jackson ImmunoResearch 703-605-155
Donkay α-Mouse 647 Jackson ImmunoResearch 715-606-150

Validation

Information below highlights manufacturer's statements as well as corresponding identification numbers for The Antibody Registry, where references for each antibody can be found.

Keratin 8 Rat DHSB TROMA-1c RRID:AB_531826
The manufacturer reports positive reactivity with mouse with recommended use in immunohistochemistry (https://dshb.biology.uiowa.edu/TROMA-I).

Keratin 14 - FITC Mouse Millipore Sigma CBL197F Clone LL002 RRID:AB_93327
The manufacturer states the antibody "is validated for use in IH(P) for the detection of cytokeratin 14" (https://www.emdmillipore.com/US/en/product/Anti-Cytokeratin-14-Antibody-clone-LL002-FITC-conjugated,MM_NF-CBL197F?ReferrerURL=https%3A%2F%2Fwww.google.com%2F#anchor_REF).

Keratin 14 Rabbit Biolegend 905301 Clone Poly19053 RRID:AB_2565048
The manufacturer reports an intended use for IHC in human tissues (https://www.biolegend.com/en-us/products/keratin-14-polyclonal-antibody-purified-10953?GroupID=GROUP26). For use in IHC for mouse tissue, see PMID: 31562298.

Keratin 5 Rabbit Biolegend 905501 Clone Poly19055 RRID:AB_2565050
The manufacturer reports an intended use for IHC in human tissues (https://www.biolegend.com/en-us/products/keratin-14-polyclonal-antibody-purified-10953?GroupID=GROUP26). For use in IHC for mouse tissue, see PMID: 31562298, 29587145, 29103953, 31631837, 30523786.

Phospho-LATS1/2 (Thr1079/1041) Rabbit Assay BioTech A8125 RRID:AB_10686029
The manufacturer reports that this antibody is validated for IHC and reactive for mouse (https://www.assaybiotechnology.com/LATS1-2-Phospho-Thr1079-1041-Antibody-A8125-IHC-ELISA). We have also previously validated this antibody for immunofluorescence in Lats1/2 depletion and phosphatase treatment experiments in mouse cells (PMID: 26235047).

YAP/TAZ Rabbit CST 8418 RRID:AB_10950494
The manufacturer reports positive reactivity with mouse in immunoprecipitation and western blot. For use in IHC, see PMID: 28867486, 28943241, 31801083. We have also validated this antibody for immunofluorescence in Yap-deleted mouse tissues in prior work (PMID: 33903236, 33903236).

GFP/YFP Chicken Aves Labs, Inc. GFP-1020 RRID:AB_10000240
The manufacturer demonstrated validation of this antibody in IHC for mouse (https://www.aveslabs.com/products/anti-green-fluorescent-protein-antibody-gfp).

GFP/YFP Rabbit Takara Bio Clontech 632592 RRID:AB_2336883
Manufacturer validation of this antibody was performed using HEK293 cells transfected with GFP (https://www.takarabio.com/documents/Certificate%20of%20Analysis/632592/632592-632593-080720.pdf). For use in IHC for mouse, see PMID: 28709801.

ERα Rabbit Abcam Ab32063 Clone E115 RRID:AB_732249
The manufacturer validated this antibody in IHC for human tissue and states reactivity with mouse (https://www.abcam.com/estrogen-receptor-alpha-antibody-e115-chip-grade-ab32063.html). Prior studies have successfully utilized this antibody for IHC in mouse tissue (PMID: 32059806, 29264547).

PR Rabbit Santa Cruz SC-538 RRID:AB_632263
The manufacturer recommends use of this antibody for detection of mouse PR via IHC (https://datasheets.scbt.com/sc-538.pdf).

Sox9 Goat R&D Systems AF3075 RRID:AB_2194160
The manufacturer reports specificity for human Sox9 in direct ELISAs and western blots (https://www.rndsystems.com/products/human-sox9-antibody_af3075#product-details). For use in IHC on mouse tissue, see PMID: 28323616, 30944105, 29587145,

29587142, 26235047. We have also validated this antibody in the experiments presented in our study.

Sox9 Rabbit Millipore-Sigma AB5535 RRID:AB_2239761
The manufacturer reports positive reactivity with mouse in IHC (https://www.emdmillipore.com/US/en/product/Anti-Sox9-Antibody,MM_NF-AB5535#anchor_REF). We have also validated this antibody in the experiments presented in our study.

YAP Rabbit CST D8H1X RRID:AB_2650491
The manufacturer reports positive reactivity with mouse in IHC (https://www.cellsignal.com/products/primary-antibodies/yap-d8h1x-xp-rabbit-mab/14074). We have also validated this antibody for immunofluorescence in YAP-deleted mouse tissues in prior work (PMID: 33903236, 33903236)

TAZ Rabbit CST E8E9G RRID:AB_2800026
The manufacturer reports reactivity with mouse in western blot, immunoprecipitation, immunofluorescence, and ChIP (https://www.cellsignal.com/products/primary-antibodies/taz-e8e9g-rabbit-mab/83669). A study has demonstrated use of this antibody in human tissue (PMID: 33232824). We have also validated this antibody for immunofluorescence in Wwtr1/TAZ-deleted mouse tissues in prior work (PMID: 33903236, 33903236).

tdTomato Goat Sicgen AB8181-200 RRID:AB_2722750
The manufacturer validated this antibody using HEK293 cells transfected with tdTomato, as well as brain sections (https://www.sicgen.pt/product/tdtomato-polyclonal-antibody_1_135). Also see PMID: 29233556, 29500351, 32134385 for further use in mouse tissues for IHC.

PCNA Mouse CST PC10 RRID:AB_2160343
The manufacturer reports reactivity with human in immunohistochemistry. We have previously used this antibody in immunofluorescence in mouse tissue (PMID:28492365).

CD45 – APC/Fire Rat Biolegend 103153 Clone 30-F11 RRID:AB_2572115 and AB_2572116
The manufacturer reports verified reactivity with mouse and previous studies have used this antibody in flow cytometry with mouse cells (PMID: 30540942, 32961131).

CD31 – APC/Fire Rat Biolegend 102433 Clone 390 RRID:AB_2629682 and AB_2629683
The manufacturer reports verified reactivity with mouse and this antibody has been previously used in flow cytometry with mouse cells (PMID: 32302523).

Ter119 – APC/Cy7 Rat Biolegend 116223 Clone TER-119 RRID:AB_2137788
The manufacturer reports verified reactivity with mouse and previous studies have used this antibody in flow cytometry with mouse cells (PMID: 31080134, 29249691).

EpCAM – Alexa 647 Rat Biolegend 118211 Clone G8.8 RRID:AB_1134101 and AB_1134104
The manufacturer reports verified reactivity with mouse and previous studies have used this antibody in flow cytometry with mouse mammary cells (PMID: 30174241, 30089273, 31597106).

CD49f – PE/Cy7 Rat Biolegend 313621 Clone GoH3 RRID:AB_2561704 and AB_2561705
The manufacturer reports documented reactivity with mouse and previous studies have used this antibody in flow cytometry with mouse mammary cells (PMID: 32697984, 32895290).

CD49b – PE Hamster Biolegend 103506 Clone Hma2 RRID:AB_313029
The manufacturer reports verified reactivity with mouse and previous studies have used this antibody in flow cytometry with mouse cells (PMID: 29727682, 28636955).

Sca-1 – BV421 Rat Biolegend 108127 Clone D7 RRID:AB_10898327 and AB_2563064
The manufacturer reports verified reactivity with mouse and previous studies have used this antibody in flow cytometry with mouse cells (PMID: 31130381, 31279774).

Animals and other organisms

Policy information about studies involving animals; ARRIVE guidelines recommended for reporting animal research

Laboratory animals	Mus musculus Lats1tm1.1Jfm/RjoJ The Jackson Laboratory JAX:024941<br>Mus musculus Lats2tm1.1Jfm/RjoJ The Jackson Laboratory JAX:025428<br>Mus musculus STOCK Tg(Krt8-cre/ERT2)17Blpn/J The Jackson Laboratory JAX:017947<br>Mus musculus B6.129X1-Gt(ROSA)26Sortm1(EYFP)Cos/J The Jackson Laboratory JAX:006148<br>Mus musculus Wwtr1tm1Hmc Yap1tm1Hmc/WranJ The Jackson Laboratory JAX:030532<br>Mus musculus Sox9-Cre (B6.129S7-Sox9<tm1(cre/ERT2)Haak>) RIKEN RBRC05522<br>Mus musculus B6.129S7-Sox9tm2Crm/J The Jackson Laboratory JAX:013016<br>Mus musculus B6.Cg-Gt(ROSA)26Sortm14(CAG-tdTomato)Hze/J The Jackson Laboratory JAX:007914<br><br>All mice used were nulliparous female mice 3-10 months of age upon initiation of experiments.
Wild animals	None were used.
Field-collected samples	None were used.
Ethics oversight	All animal experiments were done in accordance with protocols approved by the Institutional Animal Care and Use Committee at

Ethics oversight	Boston University.

Note that full information on the approval of the study protocol must also be provided in the manuscript.

Flow Cytometry

Plots

Confirm that:

☒ The axis labels state the marker and fluorochrome used (e.g. CD4-FITC).

☒ The axis scales are clearly visible. Include numbers along axes only for bottom left plot of group (a 'group' is an analysis of identical markers).

☒ All plots are contour plots with outliers or pseudocolor plots.

☒ A numerical value for number of cells or percentage (with statistics) is provided.

Methodology

Sample preparation	Immediately upon excision, the third, fourth, and fifth mammary glands were chopped with a McIlwain tissue chopper (Ted Pella, Inc.) prior to being digested at 37°C with a collagenase solution. The collagenase solution contained 2 mg/mL collagenase (Roche, 11088793001 and Worthington, LS004196), 0.5 units/mL dispase (Corning, 354235), 10 µg/mL DNAse (Roche, 4716728001), and 1x Penicillin-Streptomycin (Corning, 30-002-CI) diluted in DMEM (Corning, 10-013-CV). Red blood cells were lysed using ACK Lysing Buffer (Gibco, A1049201). Samples were dissociated to single cells using TrypLE (Gibco, 12604-021), quenched with DMEM/10% FBS, and washed with PBS. Cells were then put through a 40 µM (Fisher, 22-363-547) or 70 µM strainer (Corning, 352350). For mammary gland profiling, live/dead staining was performed using a near-IR dead cell stain (Invitrogen, L34976), followed by blocking using Fc block (BD Biosciences, 553142) in BD stain buffer (BD, 554656). Samples were then incubated in primary antibodies at the specified concentrations for 30 minutes at 4°C, washed twice, and resuspended in PBS. Compensation was performed using UltraComp eBeads Compensation Beads (Invitrogen). For FACS experiments, live/dead staining was performed using Calcein blue (Invitrogen, C1429) at a concentration of 1 µM.
Instrument	BD LSR II SORP, Beckman Coulter MoFlo Astrios
Software	FlowJo v10.7.1 (BD Biosciences), FACS Diva v6.2.1 (BD Biosciences)
Cell population abundance	All sorted cells were of interest and utilized in downstream experiments. Accuracy of the EYFP+ sorts was validated via RNA-sequencing quality control by showing observation of the expected genetic alterations in the transgenic mouse models.
Gating strategy	For mammary gland profiling experiments, cells were first gated for singlets using FSC/SSC, then gated on live cells that were CD45-CD31-TER119-. Of these, EYFP+ luminal cells were then selected using a gate on EYFP and on EpCAM with CD49f. The resultant populations were then gated on Sca1. All gates were drawn by hand based on prior literature and using reasonable boundaries observed between populations.<br><br>For FACS experiments, cells were gated on singlets using FSC and SSC, followed by gating on calcein blue for live cells. Of the live cells, EYFP+ cells were then selected to collect. All gates were drawn by hand.

☒ Tick this box to confirm that a figure exemplifying the gating strategy is provided in the Supplementary Information.
